# Joint associations of obesity and estimated GFR with clinical outcomes: a population-based cohort study

**DOI:** 10.1186/s12882-019-1351-9

**Published:** 2019-06-06

**Authors:** Marcello Tonelli, Natasha Wiebe, Csaba P. Kovesdy, Matthew T. James, Scott W. Klarenbach, Braden J. Manns, Brenda R. Hemmelgarn, for the Alberta Kidney Disease Network

**Affiliations:** 1 Department of Medicine, University of Calgary, 7th Floor, TRW Building, 3280 Hospital Drive NW, Calgary, Alberta T2N 4Z6 Canada; 2 Department of Medicine, University of Alberta, Edmonton, Alberta Canada; 3 College of Medicine, University of Tennessee Health Science Center, Memphis, TN USA

**Keywords:** eGFR, Albuminuria, Obesity

## Abstract

**Background:**

Despite the interrelationships between obesity, eGFR and albuminuria, few large studies examine how obesity modifies the association between these markers of kidney function and adverse clinical outcomes.

**Methods:**

We examined the joint associations between obesity, eGFR and albuminuria on four clinical outcomes (death, end-stage renal disease [ESRD], myocardial infarction [MI], and placement in a long-term care facility) using a population-based cohort with procedures from Alberta. Obesity was defined by body mass index ≥35 kg/m^2^ as defined by a fee modifier applied to an eligible procedure.

**Results:**

We studied 1,293,362 participants, of whom 171,650 (13.3%) had documented obesity (BMI ≥ 35 kg/m^2^ based on claims data) and 1,121,712 (86.7%) did not. The association between eGFR and death was J-shaped for participants with and without documented obesity. After full adjustment, obesity tended to be associated with slightly lower odds of mortality (OR range 0.71–1.02; p for interaction between obesity and eGFR 0.008). For participants with and without obesity, the adjusted odds of ESRD were lowest for participants with eGFR > 90 mL/min*1.73m^2^ and increased with lower eGFR, with no evidence of an interaction with obesity (*p* = 0.37). Although albuminuria and obesity were both associated with higher odds of ESRD, the excess risk associated with obesity was substantially attenuated at higher levels of albuminuria (p for interaction 0.0006). The excess risk of MI associated with obesity was observed at eGFR > 60 mL/min*1.73m^2^ but not at lower eGFR (p for interaction < 0.0001). Participants with obesity had a higher adjusted likelihood of placement in long-term care than those without, and the likelihood of such placement was higher at lower eGFR for those with and without obesity (p for interaction = 0.57).

**Conclusions:**

We found significant interactions between obesity and eGFR and/or albuminuria on the likelihood of adverse outcomes including death and ESRD. Since obesity is common, risk prediction tools for people with CKD might be improved by adding information on BMI or other proxies for body size in addition to eGFR and albuminuria.

**Electronic supplementary material:**

The online version of this article (10.1186/s12882-019-1351-9) contains supplementary material, which is available to authorized users.

## Significance statement

**What was previously known?** Obesity and CKD are both common in the general population. People with CKD and obesity have lower adjusted mortality and a higher risk of kidney failure compared to otherwise similar people without obesity.

**What were the most important findings?** The excess risk of death at lower eGFR was modestly lower in people with obesity than in otherwise similar people without obesity. In contrast, the excess risk of placement in long-term care associated with lower eGFR was modestly greater among people with obesity than without obesity, and the excess risk of progression to kidney failure among people with more severe albuminuria was attenuated in those who also had obesity.

**Why is the new information significant?** Since obesity is common in the general population, tools that use eGFR and/or albuminuria to estimate prognosis in people with CKD might benefit from including information on BMI or other proxies for body size.

## Background

Obesity is increasing in prevalence worldwide [1] and is correlated with an increased risk of various health outcomes including death, progression to end-stage renal disease (ESRD), and myocardial infarction [2, 3]. Lower estimated glomerular filtration rate (eGFR) and more severe albuminuria are also associated with these adverse outcomes [4], and people with obesity appear to have an increased risk of chronic kidney disease, including albuminuria and ESRD [5–8]. However, obesity has been associated with favorable outcomes in a number of chronic diseases, including coronary disease, heart failure and stroke [9–11]. Despite these interrelationships between obesity, eGFR and albuminuria, few large studies examine the joint associations between obesity, markers of kidney function and adverse clinical outcomes. This information is potentially important because current tools for estimating prognosis in people with CKD generally do not include information on the presence or absence of obesity [12].

We did this large population-based study of more than 1 million people treated in a universal healthcare system to examine the joint associations between obesity, eGFR and albuminuria on four clinical outcomes (death, ESRD, myocardial infarction [MI], and placement in a long-term care facility). Our primary goal was to determine whether the excess risk associated with low eGFR and albuminuria was increased or reduced by the simultaneous presence of obesity.

## Methods

This retrospective population-based cohort study is reported according to the STROBE guidelines [13]. The institutional review boards at the Universities of Alberta (Pro00053469) and Calgary (REB14–0884) approved this study.

Because some methodological aspects of this work are similar to those used in prior papers from our group, we have been asked by the journal to remove these details to avoid issues with copyright. Below we use citations to this prior work where possible to describe the methods used in the current study. Full details can be obtained by contacting our group by email.

### Data sources and cohort

We used the Alberta Kidney Disease Network database [14–16]; additional information on this data source is available elsewhere [17]. We assembled a cohort of adults who resided in Alberta, Canada on April 1, 2012 and followed them from April 2012 (baseline) until death, out-migration or study end (March 2015), whichever was earliest.

### Obesity status

Obesity status was classified as described previously [18]. Participants were classified as having obesity if claims for any procedure included a fee modification code indicating that obesity was present, and otherwise were classified as not having obesity.

### Outcomes

Clinical outcomes were all-cause death, progression to renal replacement therapy (RRT; initiation of chronic dialysis or pre-emptive kidney transplantation over 3 years of follow-up; see Additional file 1: Table S1 for specific ICD-9-CM and ICD-10-CA codes), acute myocardial infarction [19, 20], and placement into long-term care (LTC). RRT was determined using data from the Northern and Southern Alberta Renal programs. LTC placement was defined as in our prior work [21].

### Demographics

Demographic characteristics including rural location of residence were captured and classified as in our prior work [21].

### Comorbidities

Chronic kidney disease was captured using the single closest outpatient measurement of creatinine and albuminuria within 1 year of baseline (April 1, 2012). The glomerular filtration rate (GFR) was estimated using the Chronic Kidney Disease Epidemiology equation [22]. Measurements were categorized as follows: ≥105, 90–104, 60–89, 45–59, 30–44, 15–29, and < 15 mL/min*1.73m^2^. Participants had to have a measurement of GFR and to not be receiving RRT at baseline in order to be included in the cohort. Albuminuria (if available) was captured within 180 days of the baseline date and classified as in our prior work [21].

Comorbidities were defined using a previously published framework with 29 validated algorithms as applied to Canadian physician claims data, each of which had positive predictive values ≥70% as compared to a gold standard measure such as chart review [19]. Each participant was classified with respect to the presence or absence of these 29 chronic conditions at baseline as in our prior work [21].

### Statistical analyses

We did analyses with Stata MP 15·0 (www.stata.com). We used age-sex adjusted and fully adjusted logistic regression to determine whether obesity (as an interaction effect) modified the associations between eGFR and albuminuria and the clinical outcomes over 3 years of follow-up, and when the interaction effect was non-significant, whether obesity (as a main effect) was associated with the clinical outcomes. We also used logistic regression to determine the associations between eGFR and albuminuria (as main effects) and the clinical outcomes. We chose logistic regression because we used a fixed follow-up time, and logistic regression is robust to assumptions about the distribution of timing of events during follow-up. In the fully adjusted models, we also adjusted for social assistance, rural location, and the 29 comorbidities. Participants with eGFR < 15 at baseline were not included in the progression to RRT outcome. Because the decision to initiate dialysis or receive a kidney transplantation is potentially subjective, we did a sensitivity analysis that considered sustained eGFR < 10 mL/min*1·73m^2^ in addition to the initiation of RRT, as in our prior work [23]. Participants with sustained eGFR < 10 at baseline were not included in this sensitivity analysis. In a sensitivity analysis, we compared results from Cox regression to Fine and Gray [24] regression in order to determine whether death was an important competing risk for clinical outcomes. Cox regression was used for this sensitivity analysis because competing risk extensions to logistic regression are not readily available.

## Results

### Characteristics of study participants

Participant flow is shown in Additional file 1: Figure S1; 1,365,781 (39.7%) individuals were excluded since they did not have an eligible procedure and 780,809 (22.7%) were excluded because they were already on RRT or they did not have an eGFR measurement, leaving 1,293,362 participants in the cohort, of whom 171,650 (13.3%) had documented obesity and 1,121,712 (86.7%) did not. Characteristics of the people that were excluded are shown in Additional file 1: Table S2.

Over 3 years of follow-up, 48,186 of the 1,293,362 participants (3.7%) died, 1614 (0.1%) initiated renal replacement, 11,174 (0.9%) experienced a myocardial infarction and 17,785 (1.4%) were placed into long-term care. The likelihood of these four clinical outcomes without regard to obesity status is shown as a function of eGFR in Additional file 1: Figure S2 (panels A-D) and as a function of albuminuria in Additional file 1: Figure S3 (panels A-D).

### Characteristics of study participants by obesity status

Baseline characteristics of the participants by obesity status are shown in Table 1. Participants with documented obesity were more likely to be female, to receive social assistance, or to reside in a rural location than patients without documented obesity. The documented prevalence of nearly all comorbidities was higher among people with obesity than without, except for hepatitis B virus infection, dementia and Parkinson’s disease. Prevalence of lymphoma, inflammatory bowel disease and peptic ulcer disease were similar.

**Table 1:** Demographics and clinical characteristics by obesity status

Characteristics	Obesity	No obesity
N	171,650	1,121,712
Age, y		
18–39	27.4	23.5
40–64	48.3	50.1
65–79	20.5	18.8
≥80	3.8	7.7
Female	68.8	56.9
Social assistance	4.9	3.0
Rural residence location	13.4	9.1
Long-term care	1.7	1.6
eGFR, mL/min*1.73m^2^		
≥ 105	25.1	21.0
90–104	26.6	27.7
60–89	37.7	42.2
45–59	6.7	6.1
30–44	2.8	2.2
15–29	0.9	0.6
<15	0.2	0.1
Albuminuria		
Missing	21.0	23.1
Mild/none	87.3	91.8
Moderate	9.6	6.4
Severe	2.8	1.7
Morbidity		
Alcohol misuse	3.2	2.9
Asthma	6.5	2.6
Atrial fibrillation	5.6	4.1
Lymphoma	0.5	0.5
Metastatic cancer	1.2	0.9
Single site cancer	3.9	3.3
Chronic heart failure	6.7	3.8
Chronic pain	19.7	12.8
Chronic pulmonary	16.3	10.8
Chronic hepatitis B infection	0.1	0.2
Cirrhosis	0.4	0.2
Severe constipation	1.7	1.3
Dementia	1.4	2.1
Depression	16.5	11.0
Diabetes	25.2	12.0
Epilepsy	2.1	2.0
Hypertension	50.6	34.5
Hypothyroid	14.6	11.2
Inflammatory bowel disease	1.6	1.6
Irritable bowel syndrome	3.5	2.6
Multiple sclerosis	1.0	0.9
Myocardial infarction	3.0	2.4
Parkinson’s	0.6	0.7
Peptic ulcer disease	0.2	0.2
Peripheral arterial disease	1.6	1.2
Psoriasis	1.2	0.8
Rheumatoid arthritis	3.0	2.5
Schizophrenia	1.5	1.1
Stroke or transient ischemic attack	7.1	6.3

The prevalence of albuminuria was significantly higher in participants with documented obesity and diabetes and while median eGFR was similar in participants with and without documented obesity (91 vs 89 mL/min*1.73m^2^), there was broader distribution of eGFR values in participants with documented obesity, with proportionally more participants in both the left and right tails of the distribution for those with obesity. Participants with obesity were also more likely to have albuminuria assessed. The distributions of eGFR and albuminuria as a function of obesity (panels A and B) and as a function of both obesity and diabetes status (panels C-F) are shown in Additional file 1: Figure S4 for those in whom these characteristics were measured.

### Risk of mortality by obesity status

**Fig. 1:**
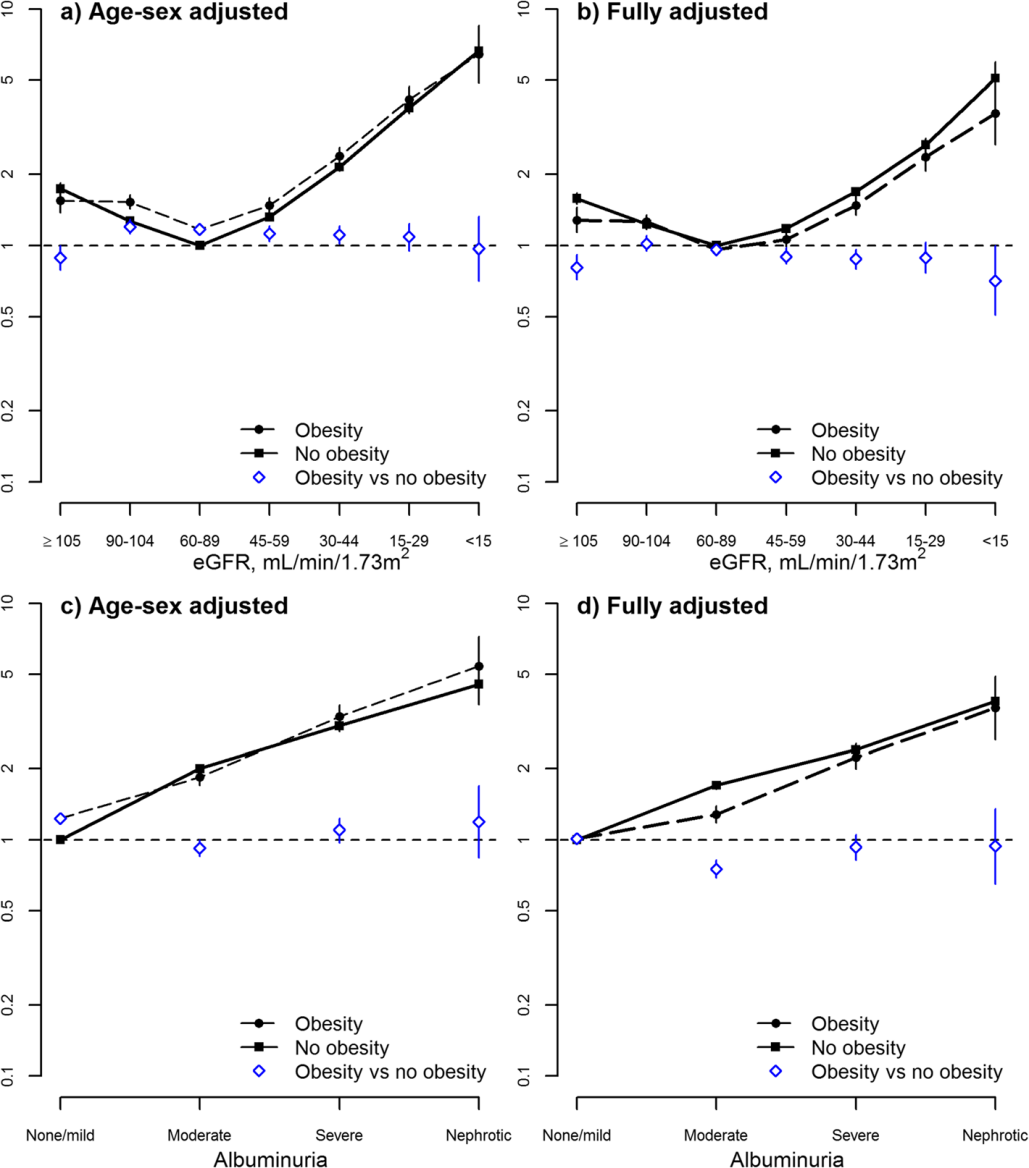
Associations of glomerular filtration rates and albuminuria with mortality, odds ratio and 95% confidence limits by documented obesity. eGFR estimated glomerular filtration rate. The top-left panel **a**) shows the age-sex adjusted association of glomerular filtration rates with mortality by documented obesity (interaction term *P* = 0.002). The top-right panel **b**) shows the fully adjusted association of glomerular filtration rates with mortality by documented obesity (interaction term *P* = 0.008). The bottom-left panel **c**) shows the age-sex adjusted association of albuminuria with mortality by documented obesity (interaction term *P* < 0.0001). The bottom-right panel **d**) shows the fully adjusted association of albuminuria with mortality by documented obesity (interaction term *P* < 0.0001)

The association between eGFR and death was J-shaped. Statistical tests for interaction between obesity and eGFR on the odds of death were significant for both age-sex-adjusted (*p* = 0.002) and fully adjusted (*p* = 0.008) models. In age-sex-adjusted analyses, the presence of documented obesity was associated with slightly higher odds of mortality in most eGFR categories (OR range 0.89–1.20, Fig. 1a). However, after full adjustment the opposite findings emerged: documented obesity tended to be associated with slightly lower odds of mortality (OR range 0.71–1.02) as compared to the absence of obesity; differences between obesity and the absence of obesity were statistically significant at eGFR < 45 and ≥ 105 mL/min*1.73m^2^ (Fig. 1b).

As expected, increasing levels of albuminuria were associated with greater odds of death (Fig. 1c, d). Statistical tests for interaction between obesity and albuminuria on the odds of death were significant for both age-sex-adjusted and fully adjusted models (*p* < 0.0001), although the magnitude of the effect modification was generally modest. The presence of documented obesity was not significantly associated with greater mortality for any albuminuria category.

### Risk of kidney failure by obesity status

**Fig. 2:**
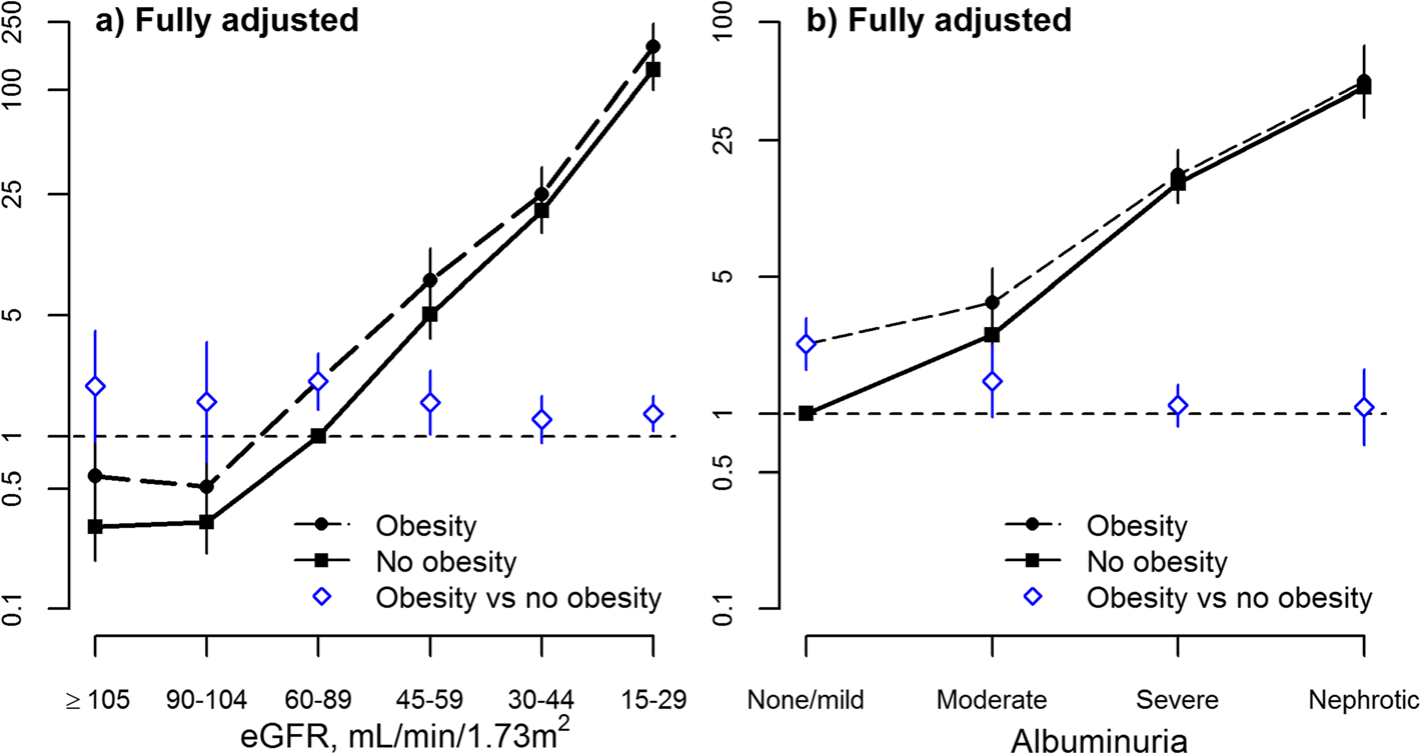
Associations of glomerular filtration rates and albuminuria with progression to RRT, odds ratio and 95% confidence limits by documented obesity. eGFR estimated glomerular filtration rate, RRT renal replacement therapy. The left panel **a**) shows the fully adjusted association of glomerular filtration rates with progression to RRT by documented obesity (interaction term *P* = 0.10). The right panel **b**) shows the fully adjusted association of albuminuria with progression to RRT by documented obesity (interaction term *P* = 0.37)

The adjusted association between eGFR and ESRD did not show convincing evidence of a J-shape. For participants with and without documented obesity, the adjusted odds of ESRD were lowest for participants with eGFR > 90 mL/min*1.73m^2^ and increased thereafter (Fig. 2a). A test for interaction between obesity and eGFR on the odds of ESRD was non-significant (*p* = 0.37); after removing the interaction term, the main effect for obesity on the odds of ESRD was OR 1.47 (95% CI 1.26, 1.72) across the full range of eGFR.

In contrast, there was a significant interaction between obesity and albuminuria on the fully adjusted odds of ESRD (p for interaction 0.0006). Although albuminuria and obesity were both associated with higher odds of ESRD, the excess risk associated with obesity was substantially attenuated at higher levels of albuminuria (Fig. 2b).

Results were similar in a sensitivity analysis that evaluated the composite outcome of ESRD or sustained eGFR < 10 mL/min*1.73m^2^. Specifically, the odds of this outcome were greater among people with obesity and with lower eGFR (or more severe albuminuria), and an interaction with obesity on the adjusted odds of death was observed for albuminuria (p for interaction 0.005) but not eGFR (p for interaction 0.12).

In a sensitivity analysis, results examining the joint relations between obesity and eGFR (and obesity and albuminuria) on the risk of ESRD were similar when Fine and Gray regression was used, as compared to Cox regression – suggesting that the competing risk of death did not materially influence the interaction between obesity and eGFR on the risk of ESRD (Additional file 1: Table S3).

### Risk of other clinical outcomes by obesity status

**Fig. 3:**
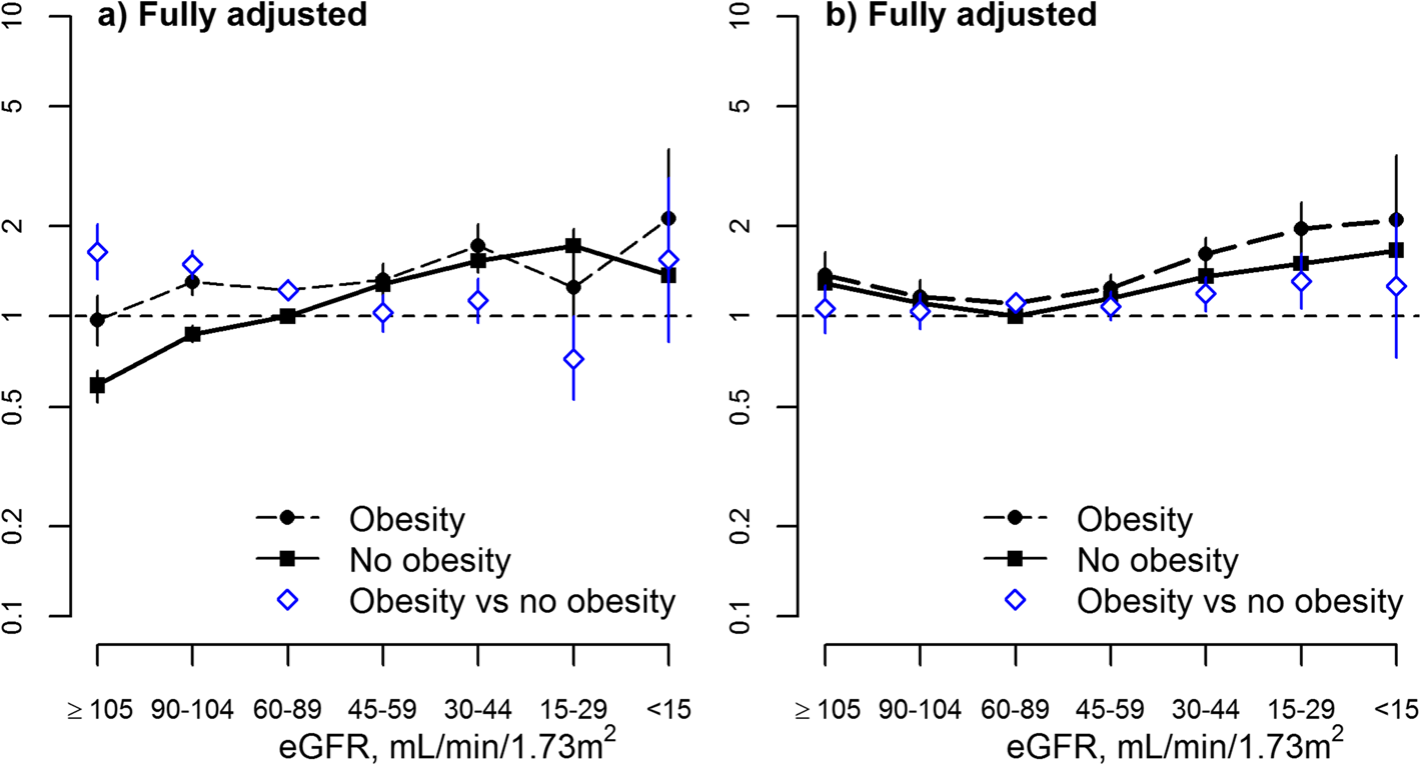
Associations of glomerular filtration rates with myocardial infarction and placement in long-term care, odds ratio and 95% confidence limits by documented obesity. eGFR estimated glomerular filtration rate. The left panel **a**) shows the fully adjusted association of glomerular filtration rates with myocardial infarction by documented obesity (interaction term *P* < 0.0001). The right panel **b**) shows the fully adjusted association of glomerular filtration rates with placement in long-term care by documented obesity (interaction term *P* < 0.0001)

As expected, the odds of myocardial infarction were higher at lower eGFR. There was a significant interaction between obesity and eGFR on the fully adjusted odds of myocardial infarction (p for interaction < 0.0001), such that an excess risk of MI associated with obesity was observed at eGFR > 60 mL/min*1.73m^2^ but not at lower eGFR (Fig. 3a).

Participants with documented obesity had a significantly higher adjusted likelihood of placement in long-term care than those without, and the odds of such placement was higher at lower eGFR. There was no evidence of a significant interaction between obesity and eGFR on the fully adjusted odds of placement in long-term care (p for interaction = 0.57). After removing the interaction term, the main effect for obesity on the risk of placement in a long-term care facility was OR 1.11 (95% CI 1.06, 1.17) across the full range of eGFR (Fig. 3b).

Results from Cox regression were again similar to those from Fine and Gray regression in analyses examining the joint associations of obesity and eGFR (and obesity and albuminuria) on the odds of myocardial infarction and the odds of placement in long-term care.

## Discussion

In this observational study of more than 1 million people, we examined the joint associations between markers of kidney disease (eGFR, albuminuria) and obesity with a range of clinical outcomes including death, ESRD, MI, and placement in a long-term care facility. In all analyses, obesity was defined by BMI ≥35 kg/m^2^ as defined by a fee modifier applied to an eligible procedure. There was a significant interaction between obesity and eGFR on the odds of all-cause mortality – after adjustment for potential confounders, the excess risk of death at lower eGFR was modestly lower in people with obesity than in otherwise comparable people without obesity. A similar interaction between obesity and albuminuria was seen on the odds of ESRD -- the excess risk of progression to kidney failure among people with more severe albuminuria was slightly attenuated in those who also had obesity. In contrast, the excess risk of placement in long-term care associated with lower eGFR was greater among people with obesity than without obesity. Finally, although the risk of myocardial infarction appeared to be modestly greater at lower eGFR for both people with and without obesity, there was no evidence that obesity (as compared to the absence of obesity) was associated with an excess risk of myocardial infarction among people with eGFR < 60 mL/min*1.73m^2^.

Although others have reported that people with CKD and obesity have lower adjusted mortality compared to otherwise similar people without obesity [25, 26], we are not aware of prior studies suggesting that obesity may attenuate the higher adjusted risk of death associated with CKD. The latter finding is consistent with the hypothesis that obesity acts as a potential buffer against the risk of death associated with acute illnesses, which in turn may be more common at lower eGFR or with more severe albuminuria. Other potential explanations for the apparent protective effects of obesity in CKD have been described elsewhere [3], and include neutralization of uremic toxins or harmful cytokines by adipose tissue, enhanced antioxidant status, the inclusion of chronically ill (underweight) people in the comparator group (classified as not having obesity), and a more favorable hemodynamic response to stressful stimuli.

Similarly, although faster kidney function loss among people with higher vs lower BMI has been previously reported [7, 8, 27], we are not aware of other work demonstrating that obesity attenuates the excess risk of kidney failure associated with more severe albuminuria. Results were consistent in a sensitivity analysis that used a more objective definition of kidney failure (initiation of dialysis or sustained eGFR < 10 mL/min*1.73m^2^), reducing the possibility that this finding was due to bias resulting from differential initiation of dialysis among people with vs without obesity. Using a model that explicitly accounted for competing risks (Fine and Gray regression) did not change results, suggesting that differential mortality between groups does not explain our findings.

We found that obesity also accentuates the known association between lower eGFR and the likelihood of placement in a long-term care facility [21], perhaps because obesity may further impede activities of daily living among people who already have reduced functional status due to chronic illness. The lack of an association between obesity and the adjusted risk of myocardial infarction among people with reduced eGFR (in contrast to the significant association observed among those with eGFR > 60 mL/min*1.73m^2^) may be attributable to confounding by inflammation among the subset of non-obese people with evidence of wasting and/or malnutrition, which have been associated with a higher risk of vascular disease and other adverse outcomes [28]. However, since we did not have data on inflammation, wasting or malnutrition, this suggestion is speculative.

Previous studies have consistently shown that obesity modifies the association between eGFR and the risk of death but generally did not examine how obesity interacts with albuminuria to influence the likelihood of death or other clinical outcomes. For example, prior reports indicate that obesity is associated with excess mortality in people with apparently normal kidney function, and in those with mild to moderate reductions in eGFR. Lu et al’s study of 453,946 US Veterans [26] demonstrated a significantly higher adjusted risk of death among participants with BMI ≥35 kg/m^2^ but only among those with eGFR > 30 mL/min*1.73m^2^. Below this level of eGFR, the adjusted risk of death among participants with and without obesity did not significantly differ. Differences between our study and previous work may relate to our use of a cohort drawn from the general population as compared to a subset thereof (e.g., U. S Veterans; Israeli adolescents) [8, 27], our ability to adjust for both eGFR and albuminuria, the comprehensive panel of comorbidities for which we adjusted, or greater statistical power due to the large sample size of the current cohort.

Existing tools for estimating prognosis in people with CKD incorporate information on eGFR, albuminuria, age, and other clinical characteristics – but generally do not consider obesity. Our findings suggest that the relations between eGFR, albuminuria and adverse clinical outcomes may vary by the presence of obesity, which in turn indicates that there may be value in incorporating information on BMI into these prognostic tools. Future studies should examine the incremental value of including obesity as both a main effect and an interaction term in models linking eGFR and albuminuria to the risk of clinical outcomes.

Like other reports from our group, this study has several important strengths [21]. However, our study also has certain limitations that should be considered when interpreting results. First, although the algorithms used to classify patients with respect to obesity have been previously used [18], they have not been externally validated and thus some misclassification is possible. In addition, we used a relatively crude definition of obesity (BMI ≥35 kg/m^2^), and therefore some participants with BMI between 30 and 34.9 would have been incorrectly classified as not having obesity. Second, BMI is known to be an insensitive predictor of obesity as assessed by measures of body composition among people with CKD specifically [29], and so some differential misclassification of kidney function may have occurred at lower levels of eGFR. Third, only Alberta residents who underwent an eligible procedure and had information on eGFR or albuminuria were included in the study. Thus, whether our findings are generalizable to a broader population is uncertain. Fourth, like many prior studies using administrative data, we required only a single measure of eGFR to define CKD, which might have led to misclassification of CKD status as compared to requiring at least two measurements that were > 90 days apart. Finally, although we adjusted for a panel of more than 40 potential confounders (including 32 comorbidities), we did not have data on tobacco use, dietary habits, physical activity, insulin resistance, or family history of chronic disease, and thus residual confounding by these characteristics is possible.

## Conclusion

In summary, our study suggests that obesity influences the magnitude of the excess risk for adverse outcomes (such as death and ESRD) that is associated with lower baseline eGFR or more severe albuminuria. Since obesity is common in the general population, tools that use eGFR and/or albuminuria to estimate prognosis in people with CKD might benefit from including information on BMI or other proxies for body size.

## Additional file

Additional file 1: **Table S1.** ICD-9-CM and ICD-10-CA codes for clinical outcomes. **Table S2.** Demographics and clinical characteristics by obesity status. **Table S3.** Progression to RRT by obesity where mortality is modelled as a competing risk, HR (95% CI). **Figure S1.** Participant flow diagram. **Figure S2.** Associations of glomerular filtration rates with clinical outcomes, odds ratio and 95% confidence limits. **Figure S3.** Associations of albuminuria with clinical outcomes, odds ratio and 95% confidence limits. **Figure S4.** Quantile-quantile plots of estimated glomerular filtration rate and albumin:creatinine ratio (DOCX 420 kb)

## Abbreviations

ACR: Albumin creatinine ratio
BMI: Body mass index
CKD: Chronic kidney disease
eGFR: Estimated glomerular filtration rate
ESRD: End-stage renal disease
ICD: International classification of disease
LTC: Long-term care
MI: Myocardial infarction
OR: Odds ratio
PCR: Protein creatinine ratio
RRT: Renal replacement therapy
STROBE: Strengthening the reporting of observational studies in epidemiology

## References

[1] Ng M, Fleming T, Robinson M, Thomson B, Graetz N, Margono C (2014). Global, regional, and national prevalence of overweight and obesity in children and adults during 1980-2013: a systematic analysis for the global burden of disease study 2013. Lancet..

[2] Afshin A, Forouzanfar MH, Reitsma MB, Sur P, Estep K, GBD 2015 Obesity Collaborators (2017). Health effects of overweight and Obesity in 195 countries over 25 years. N Engl J Med.

[3] Kovesdy CP, Furth SL, Zoccali C (2017). World kidney day steering C. Obesity and kidney disease: hidden consequences of the epidemic. Can J Kidney Health Dis.

[4] Hemmelgarn BR, Manns BJ, Lloyd A, James MT, Klarenbach S, Quinn RR (2010). Relation between kidney function, proteinuria, and adverse outcomes. Jama..

[5] Elsayed EF, Sarnak MJ, Tighiouart H, Griffith JL, Kurth T, Salem DN (2008). Waist-to-hip ratio, body mass index, and subsequent kidney disease and death. Am J Kidney Dis.

[6] Pinto-Sietsma SJ, Navis G, Janssen WM, de Zeeuw D, Gans RO, de Jong PE (2003). A central body fat distribution is related to renal function impairment, even in lean subjects. Am J Kidney Dis.

[7] Hsu CY, McCulloch CE, Iribarren C, Darbinian J, Go AS (2006). Body mass index and risk for end-stage renal disease. Ann Intern Med.

[8] Vivante A, Golan E, Tzur D, Leiba A, Tirosh A, Skorecki K (2012). Body mass index in 1.2 million adolescents and risk for end-stage renal disease. Arch Intern Med.

[9] Romero-Corral A, Montori VM, Somers VK, Korinek J, Thomas RJ, Allison TG (2006). Association of bodyweight with total mortality and with cardiovascular events in coronary artery disease: a systematic review of cohort studies. Lancet..

[10] Oreopoulos A, Padwal R, Kalantar-Zadeh K, Fonarow GC, Norris CM, McAlister FA (2008). Body mass index and mortality in heart failure: a meta-analysis. Am Heart J.

[11] Oesch L, Tatlisumak T, Arnold M, Sarikaya H (2017). Obesity paradox in stroke - myth or reality? A systematic review. PLoS One.

[12] Tangri N, Grams ME, Levey AS, Coresh J, Appel LJ, Astor BC (2016). Multinational assessment of accuracy of equations for predicting risk of kidney failure: a meta-analysis. Jama..

[13] von Elm E, Altman DG, Egger M, Pocock SJ, Gotzsche PC, Vandenbroucke JP (2007). The strengthening the reporting of observational studies in epidemiology (STROBE) statement: guidelines for reporting observational studies. Lancet..

[14] Thompson S, James M, Wiebe N, Hemmelgarn B, Manns B, Klarenbach S (2015). Cause of death in patients with reduced kidney function. J Am Soc Nephrol.

[15] Tonelli M, Muntner P, Lloyd A, Manns BJ, Klarenbach S, Pannu N (2012). Risk of coronary events in people with chronic kidney disease compared with those with diabetes: a population-level cohort study. Lancet..

[16] Alexander RT, Hemmelgarn BR, Wiebe N, Bello A, Morgan C, Samuel S (2012). Kidney stones and kidney function loss: a cohort study. Bmj..

[17] Hemmelgarn BR, Clement F, Manns BJ, Klarenbach S, James MT, Ravani P (2009). Overview of the Alberta kidney disease network. BMC Nephrol.

[18] Bello A, Padwal R, Lloyd A, Hemmelgarn B, Klarenbach S, Manns B (2013). Using linked administrative data to study periprocedural mortality in obesity and chronic kidney disease (CKD). Nephrology, dialysis, transplantation : official publication of the European Dialysis and transplant association. Eur Ren Assoc.

[19] Tonelli M, Wiebe N, Martin F, Guthrie B, Hemmelgarn BR, James MT (2015). Methods for identifying 30 chronic conditions: application to administrative data. BMC Med Inform Decis Mak.

[20] Austin PC, Daly PA, Tu JV (2002). A multicenter study of the coding accuracy of hospital discharge administrative data for patients admitted to cardiac care units in Ontario. Am Heart J.

[21] Tonelli M, Wiebe N, James MT, Klarenbach SW, Manns BJ, Ravani P (2018). A population-based cohort study defines prognoses in severe chronic kidney disease. Kidney Int.

[22] Stevens PE, Levin A (2013). Kidney Disease: Improving global outcomes chronic kidney disease guideline development work group M. evaluation and management of chronic kidney disease: synopsis of the kidney disease: improving global outcomes 2012 clinical practice guideline. Ann Intern Med.

[23] Tonelli M, Wiebe N, James MT, Naugler C, Manns BJ, Klarenbach SW (2019). Red cell distribution width associations with clinical outcomes: a population-based cohort study. PLoS One.

[24] Fine JP, Gray RJ (1999). A proportional hazards model for the subdistribution of a competing risk. J Am Stat Assoc.

[25] Beddhu S, Pappas LM, Ramkumar N, Samore M (2003). Effects of body size and body composition on survival in hemodialysis patients. J Am Soc Nephrol.

[26] Lu JL, Kalantar-Zadeh K, Ma JZ, Quarles LD, Kovesdy CP (2014). Association of body mass index with outcomes in patients with CKD. J Am Soc Nephrol.

[27] Lu JL, Molnar MZ, Naseer A, Mikkelsen MK, Kalantar-Zadeh K, Kovesdy CP (2015). Association of age and BMI with kidney function and mortality: a cohort study. Lancet Diabetes Endocrinol.

[28] Park J, Ahmadi SF, Streja E, Molnar MZ, Flegal KM, Gillen D (2014). Obesity paradox in end-stage kidney disease patients. Prog Cardiovasc Dis.

[29] Carrero JJ (2014). Misclassification of obesity in CKD: appearances are deceptive. Clin J Am Soc Nephrol.

