# Supplementary material for: Joint associations of obesity and estimated GFR with clinical outcomes: a population-based cohort study
Source: BMC Nephrol. 2019 Jun 6;20:204. doi: 10.1186/s12882-019-1351-9 (PMC6555725; doi:10.1186/s12882-019-1351-9)
Supplement: Supplementary file 1 — Table S1. ICD-9-CM and ICD-10-CA codes for clinical outcomes. Table S2. Demographics and clinical characteristics by obesity status. Table S3. Progression to RRT by obesity where mortality is modelled as a competing risk, HR (95% CI). Figure S1. Participant flow diagram. Figure S2. Associations of glomerular filtration rates with clinical outcomes, odds ratio and 95% confidence limits. Figure S3. Associations of albuminuria with clinical outcomes, odds ratio and 95% confidence limits. Figure S4. Quantile-quantile plots of estimated glomerular filtration rate and albumin:creatinine ratio (DOCX 420 kb) [file 12882_2019_1351_MOESM1_ESM.docx]

**Table S1. ICD-9-CM and ICD-10-CA codes for clinical outcomes**

| **Variables** | **ICD-9-CM** | **ICD-10-CA** |
| --- | --- | --- |
|  |  |  |
| Chronic dialysis | CCPx codes: 13.99A, 13.99B, 13.99C,  13.99D, 13.99O, 13.99OA | - |
| Kidney Transplantation | CCPx code: 67.5  Procedure code: 55.69 | CCI code: 1.PC.85 |
|  |  |  |

**Table S2. Demographics and clinical characteristics by obesity status**

| **Characteristics** | **Excluded**  **from cohort^1^** | **Cohort** |
| --- | --- | --- |
| N | 2,142,338 | 1,293,362 |
| Age, y |  |  |
| 18-39 | 55.6 | 24.0 |
| 40-64 | 37.8 | 49.8 |
| 65-79 | 4.8 | 19.0 |
| ≥80 | 1.9 | 7.2 |
| Female | 43.2 | 58.5 |
| Indigenous | 3.5 | 2.5 |
| Social assistance | 1.9 | 3.3 |
| Rural residence location | 8.3 | 9.7 |
| Long-term care | 0.5 | 1.6 |
| eGFR, mL/min*1.73m^2^ |  |  |
| Missing | 84.3 | 0.0 |
| ≥105 | 30.5 | 21.5 |
| 90-104 | 32.0 | 27.6 |
| 60-89 | 33.6 | 41.6 |
| 45-59 | 2.8 | 6.2 |
| 30-44 | 0.9 | 2.3 |
| 15-29 | 0.2 | 0.7 |
| <15 | 0.0 | 0.1 |
| Albuminuria |  |  |
| Missing | 82.5 | 22.8 |
| Mild/none | 93.0 | 91.2 |
| Moderate | 5.8 | 6.8 |
| Severe | 1.2 | 1.8 |
| Morbidity |  |  |
| Alcohol misuse | 2.0 | 2.9 |
| Asthma | 1.1 | 3.1 |
| Atrial fibrillation | 0.6 | 4.3 |
| Lymphoma | 0.0 | 0.5 |
| Metastatic cancer | 0.1 | 0.9 |
| Single site cancer | 0.4 | 3.4 |
| Chronic heart failure | 0.6 | 4.2 |
| Chronic pain | 3.7 | 13.7 |
| Chronic pulmonary | 3.3 | 11.6 |
| Chronic hepatitis B infection | 0.1 | 0.2 |
| Cirrhosis | 0.0 | 0.2 |
| Severe constipation | 0.2 | 1.4 |
| Dementia | 0.5 | 2.0 |
| Depression | 4.5 | 11.8 |
| Diabetes | 3.1 | 13.8 |
| Epilepsy | 0.9 | 2.0 |
| Hypertension | 9.1 | 36.6 |
| Hypothyroid | 2.9 | 11.6 |
| Inflammatory bowel disease | 0.4 | 1.6 |
| Irritable bowel syndrome | 0.7 | 2.7 |
| Multiple sclerosis | 0.3 | 0.9 |
| Myocardial infarction | 0.4 | 2.5 |
| Parkinson’s | 0.1 | 0.7 |
| Peptic ulcer disease | 0.0 | 0.2 |
| Peripheral arterial disease | 0.2 | 1.3 |
| Psoriasis | 0.3 | 0.9 |
| Rheumatoid arthritis | 0.4 | 2.5 |
| Schizophrenia | 0.7 | 1.1 |
| Stroke or transient ischemic attack | 1.4 | 6.4 |
|  |  |  |

^1^those without a procedure and those without an eGFR measurement

**Table S3. Progression to RRT by obesity where mortality is modelled as a competing risk, HR (95% CI)**

| **eGFR,**  **mL/min*1.73m^2^** | **Obesity** | **No obesity** | **Obesity vs**  **no obesity** |  |
| --- | --- | --- | --- | --- |
| *Glomerular filtration rate* | | | | |
| Fully adjusted - Cox |  |  |  |  |
| ≥105 | 0.70 (0.35,1.42) | 0.30 (0.18,0.51) | 2.29 (1.04,5.08) |  |
| 90-104 | 0.53 (0.23,1.21) | 0.34 (0.21,0.55) | 1.55 (0.63,3.86) |  |
| 60-89 | 2.18 (1.41,3.37) | 1.00 | 2.18 (1.41,3.37) |  |
| 45-59 | 8.93 (5.53,14.44) | 5.97 (4.16,8.57) | 1.50 (0.93,2.42) |  |
| 30-44 | 27.49 (18.37,41.13) | 24.19 (17.36,33.71) | 1.14 (0.80,1.61) |  |
| 15-29 | 177.60 (126.61,249.10) | 135.45 (99.66,184.09) | 1.31 (1.06,1.62) |  |
| Fully adjusted - Fine |  |  |  |  |
| ≥105 | 0.68 (0.33,1.40) | 0.30 (0.18,0.51) | 2.27 (1.02,5.04) |  |
| 90-104 | 0.52 (0.22,1.19) | 0.34 (0.21,0.55) | 1.54 (0.62,3.81) |  |
| 60-89 | 2.15 (1.39,3.35) | 1.00 | 2.15 (1.39,3.35) |  |
| 45-59 | 9.00 (5.45,14.87) | 6.01 (4.10,8.82) | 1.50 (0.93,2.42) |  |
| 30-44 | 27.53 (17.78,42.62) | 23.69 (16.30,34.44) | 1.16 (0.83,1.63) |  |
| 15-29 | 158.36 (106.54,235.39) | 123.98 (85.74,179.26) | 1.28 (1.02,1.60) |  |
| *Albuminuria* | | | | |
| Fully adjusted - Cox |  |  |  |  |
| None/mild | 2.29 (1.57,3.33) | 1.00 | 2.29 (1.57,3.33) |  |
| Moderate | 4.58 (2.89,7.27) | 2.90 (2.02,4.16) | 1.58 (0.97,2.56) |  |
| Severe | 21.36 (15.49,29.47) | 19.92 (15.12,26.25) | 1.07 (0.85,1.35) |  |
| Nephrotic | 56.93 (38.48,84.24) | 41.62 (29.55,58.62) | 1.37 (0.96,1.95) |  |
| Missing | 5.84 (3.84,8.89) | 2.88 (2.11,3.93) | 2.03 (1.32,3.11) |  |
| Fully adjusted - Fine |  |  |  |  |
| None/mild | 2.28 (1.56,3.33) | 1.00 | 2.28 (1.56,3.33) |  |
| Moderate | 4.46 (2.79,7.14) | 2.87 (1.99,4.15) | 1.55 (0.96,2.51) |  |
| Severe | 20.55 (14.47,29.18) | 19.15 (14.11,25.99) | 1.07 (0.85,1.36) |  |
| Nephrotic | 48.31 (31.49,74.11) | 36.98 (24.84,55.04) | 1.31 (0.89,1.91) |  |
| Missing | 5.59 (3.71,8.42) | 2.78 (2.04,3.79) | 2.01 (1.32,3.07) |  |
|  |  |  |  |  |

CI confidence interval, HR hazard ratio

**Figure S1. Participant flow diagram**

**
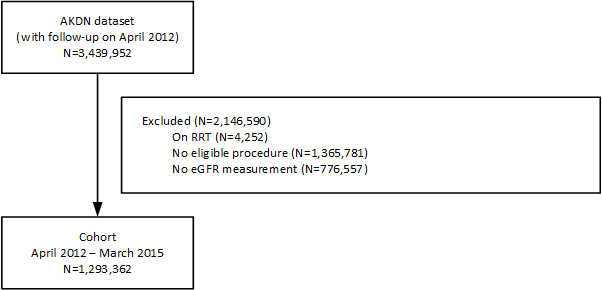
**

AKDN Alberta Kidney Disease Network

**Figure S2. Associations of glomerular filtration rates with clinical outcomes, odds ratio and 95% confidence limits**

**
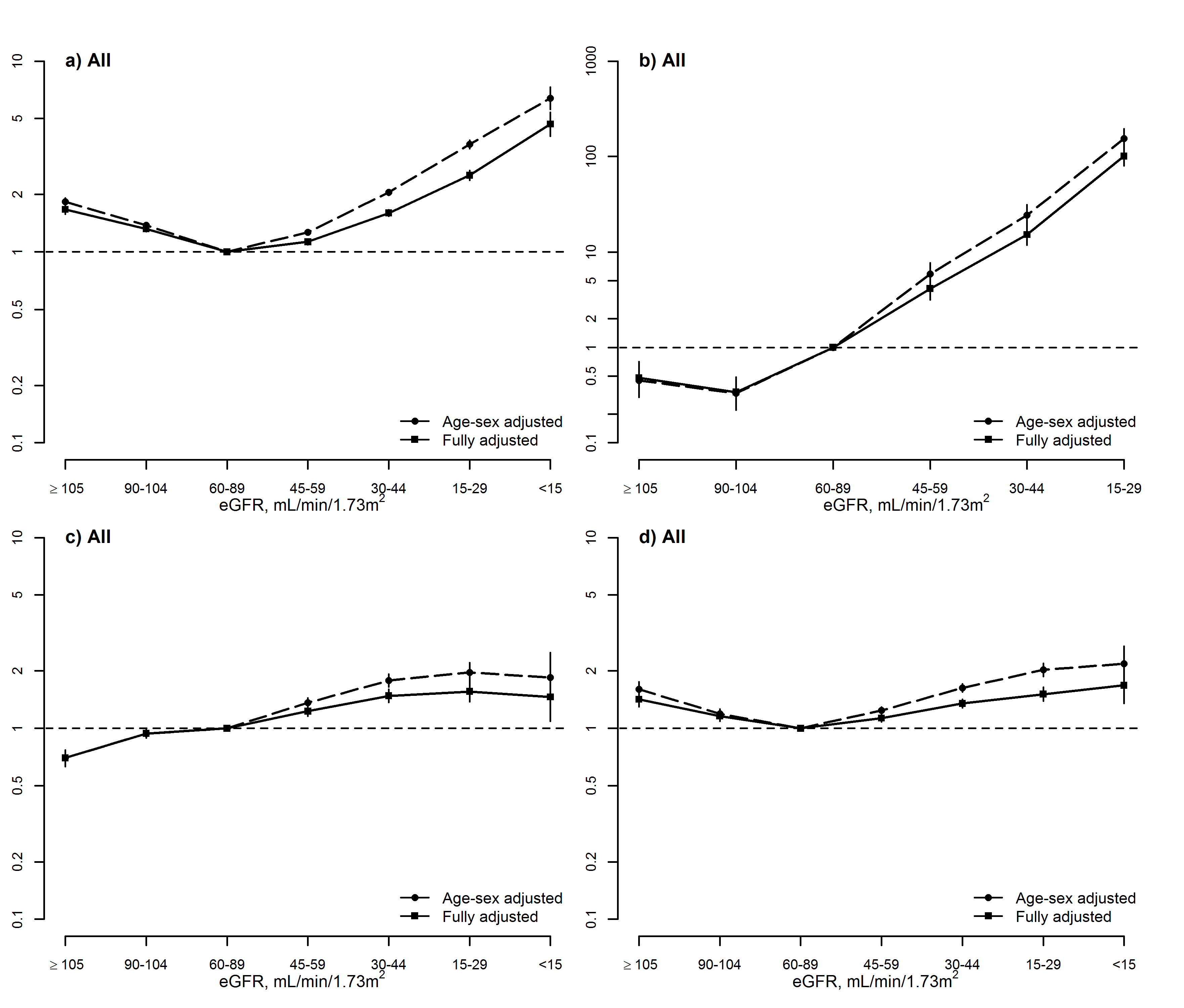
**

eGFR estimated glomerular filtration rate, RRT renal replacement therapy

The top-left panel a) shows the age-sex and fully adjusted association of glomerular filtration rates with mortality. The top-right panel b) shows the age-sex and fully adjusted association of glomerular filtration rates with progression to RRT. The bottom-left panel c) shows the age-sex and fully adjusted association of glomerular filtration rates with myocardial infarction. The bottom-right panel d) shows the age-sex and fully adjusted association of glomerular filtration rates with placement in long-term care.

**Figure S3. Associations of albuminuria with clinical outcomes, odds ratio and 95% confidence limits**

**
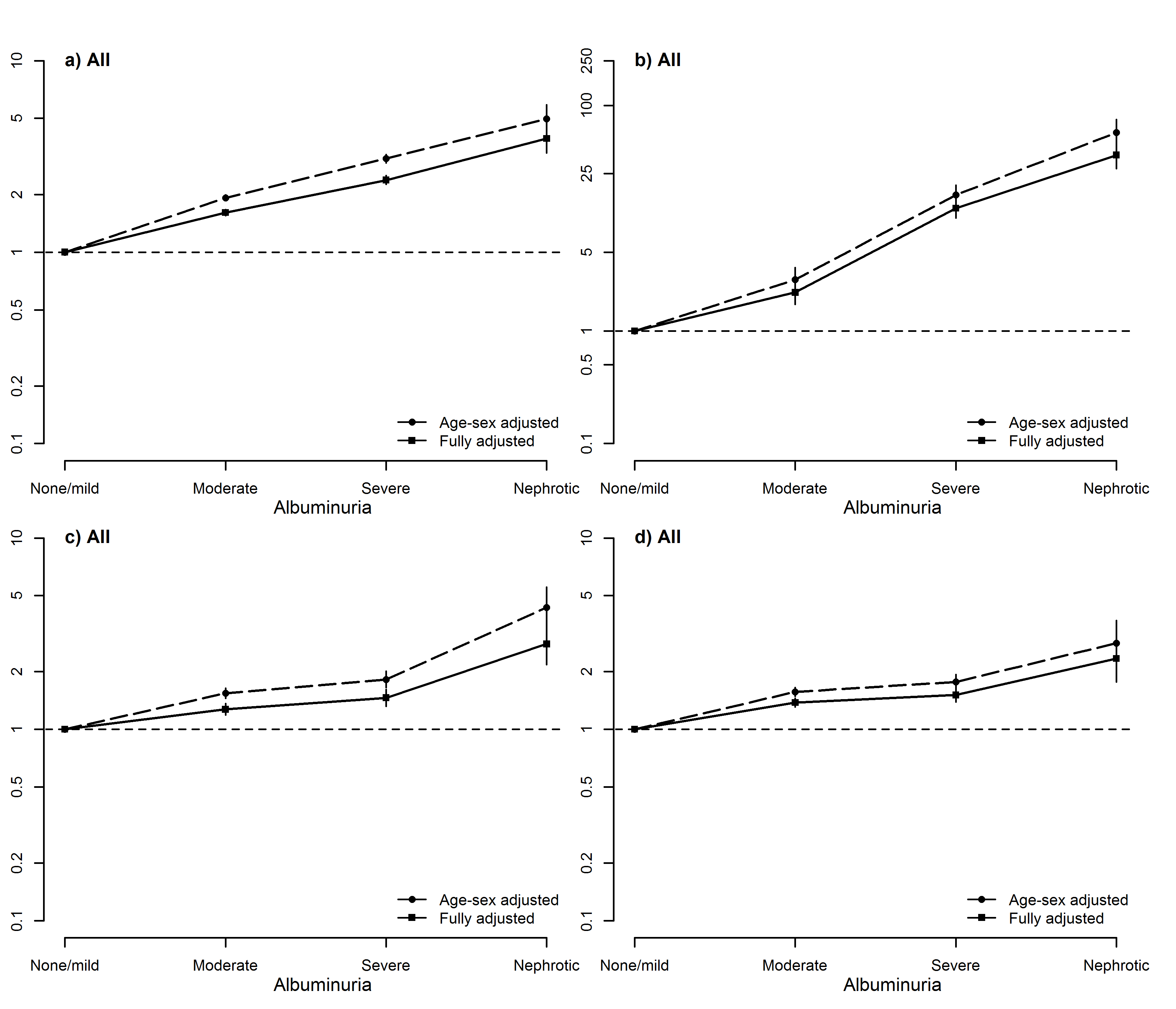
**

eGFR estimated glomerular filtration rate, RRT renal replacement therapy

The top-left panel a) shows the age-sex and fully adjusted association of albuminuria with mortality. The top-right panel b) shows the age-sex and fully adjusted association of albuminuria with progression to RRT. The bottom-left panel c) shows the age-sex and fully adjusted association of albuminuria with myocardial infarction. The bottom-right d) panel shows the age-sex and fully adjusted association of albuminuria with placement in long-term care.

**Figure S4. Quantile-quantile plots of estimated glomerular filtration rate and albumin:creatinine ratio**


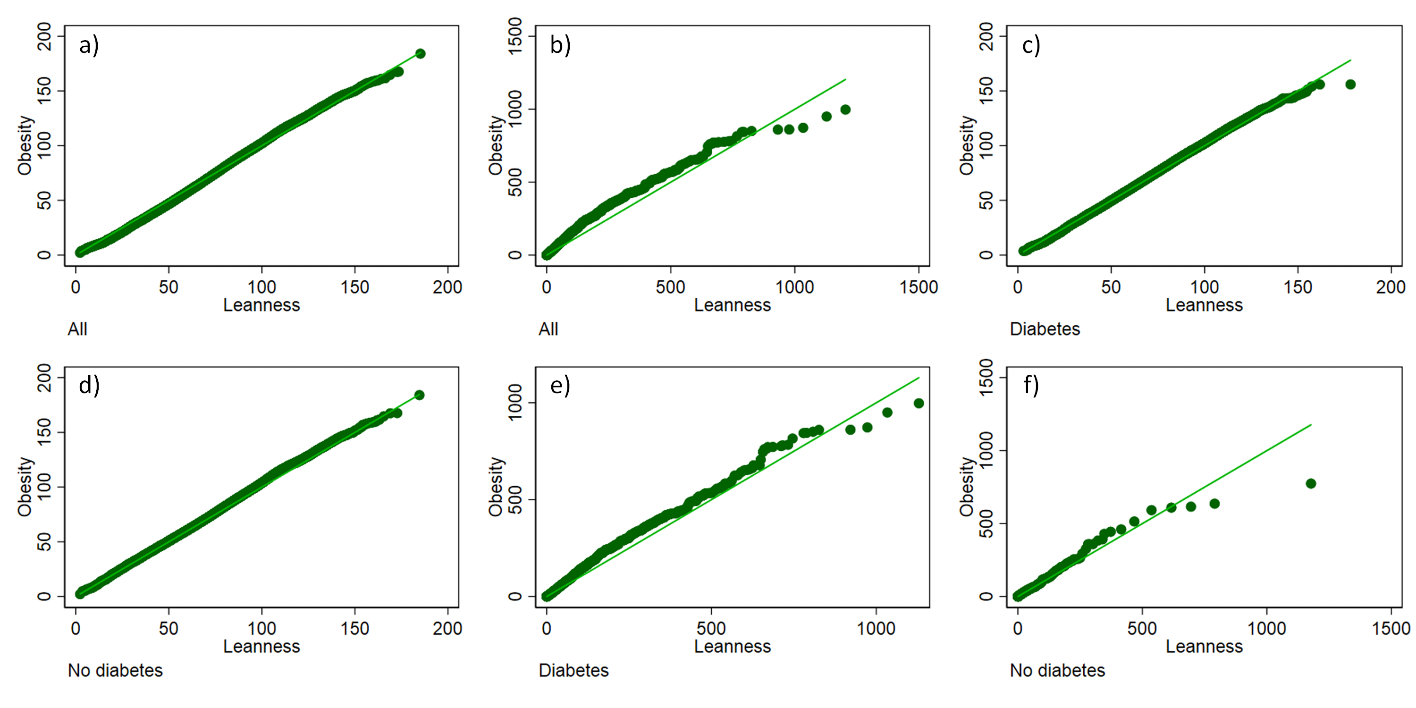


The quantile-quantile (Q-Q) plots assess whether the distributions of estimated glomerular filtration rate (eGFR) and albumin:creatinine ratio (ACR) are similar between participants with and without documented obesity. The top-left plot a) shows all participants with available eGFR measurements. The top-middle plot b) shows all participants with available ACR measurements. The top-right plot c) shows all diabetic participants (N=177,986) with available eGFR measurements. The bottom-left plot d) shows all non-diabetic participants (N=1,115,376) with available eGFR measurements. The bottom-middle plot e) shows all diabetic participants (N=93,971) with available ACR measurements and the bottom-right plot f) shows all non-diabetic participants (N=85,210) with available ACR measurements. As most of the values in the bottom right plot are above the reference line, this Q-Q plot suggests that diabetic participants with documented obesity have, on average, higher ACR measurements.
